# The Spontaneous Autoimmune Neuromyopathy in ICOSL^−/−^ NOD Mice Is CD4^+^ T-Cell and Interferon-γ Dependent

**DOI:** 10.3389/fimmu.2017.00287

**Published:** 2017-03-31

**Authors:** Claire Briet, Gwladys Bourdenet, Ute C. Rogner, Chantal Becourt, Isabelle Tardivel, Laurent Drouot, Christophe Arnoult, Jean-Claude do Rego, Nicolas Prevot, Charbel Massaad, Olivier Boyer, Christian Boitard

**Affiliations:** ^1^INSERM U1016, Cochin Institute, Paris Descartes University, Sorbonne Paris Cité, Paris, France; ^2^Normandie Université, UNIROUEN, INSERM, U1234, Rouen University Hospital, Department of Immunology, Rouen, France; ^3^INSERM U1016, Cochin Institute, Paris, France; ^4^Normandie Université, UNIROUEN, CNRS, UMR 6270, Rouen, France; ^5^Normandie Université, UNIROUEN, SCAC, INSERM, U1234, Rouen, France; ^6^Developmental Immunology, Department of Paediatrics, and the Weatherall Institute of Molecular Medicine, University of Oxford, Oxford, UK; ^7^INSERM UMR S1124, Paris Descartes University, Paris, France

**Keywords:** autoimmunity, type 1 diabetes, neuropathy, myopathy, costimulation, ICOS, ICOSL

## Abstract

Abrogation of ICOS/ICOS ligand (ICOSL) costimulation prevents the onset of diabetes in the non-obese diabetic (NOD) mouse but, remarkably, yields to the development of a spontaneous autoimmune neuromyopathy. At the pathological level, ICOSL^−/−^ NOD mice show stronger protection from insulitis than their ICOS^−/−^ counterparts. Also, the ICOSL^−/−^ NOD model carries a limited C57BL/6 region containing the *Icosl* nul mutation, but, in contrast to ICOS^−/−^ NOD mice, no gene variant previously reported as associated to NOD diabetes. Therefore, we aimed at providing a detailed characterization of the ICOSL^−/−^ NOD model. The phenotype observed in ICOSL^−/−^ NOD mice is globally similar to that observed in ICOS^−/−^ and ICOS^−/−^ICOSL^−/−^ double-knockout NOD mice, manifested by a progressive locomotor disability first affecting the front paws as observed by catwalk analysis and a decrease in grip test performance. The pathology remains limited to peripheral nerve and striated muscle. The muscle disease is characterized by myofiber necrosis/regeneration and an inflammatory infiltrate composed of CD4^+^ T-cells, CD8^+^ T-cells, and myeloid cells, resembling human myositis. Autoimmune neuromyopathy can be transferred to NOD.*scid* recipients by CD4^+^ but not by CD8^+^ T-cells isolated from 40-week-old female ICOSL^−/−^ NOD mice. The predominant role of CD4^+^ T-cells is further demonstrated by the observation that neuromyopathy does not develop in CIITA^−/−^ICOSL^−/−^ NOD in contrast to β2microglobulin^−/−^ICOSL^−/−^ NOD mice. Also, the cytokine profile of CD4^+^ T-cells infiltrating muscle and nerve of ICOSL^−/−^ NOD mice is biased toward a Th1 pattern. Finally, adoptive transfer experiments show that diabetes development requires expression of ICOSL, in contrast to neuromyopathy. Altogether, the deviation of autoimmunity from the pancreas to skeletal muscles in the absence of ICOS/ICOSL signaling in NOD mice is strictly dependent on CD4^+^ T-cells, leads to myofiber necrosis and regeneration. It provides the first mouse model of spontaneous autoimmune myopathy akin to human myositis.

## Introduction

Type 1 diabetes (T1D) is an autoimmune disease characterized by the activation of autoreactive lymphocytes against pancreatic β-cell antigens. Mechanisms that initiate the failure of immune tolerance to β-cells remain elusive in common forms of T1D (1, 2).

In animal models such as the non-obese diabetic (NOD) mouse, the predominant role of T-cells is supported by experiments in which diabetes is transferred into naive recipients by T-cells from diabetic or prediabetic animals (3). Moreover, T1D is prevented by injection of antibodies (Abs) that interfere with T-cells activation and fails to develop in diabetes-prone mice in which key genes in T-cells differentiation or activation are non-functional (4, 5). Both CD4^+^ and CD8^+^ T-cells are involved in diabetes development and both major histocompatibility complex (MHC) class I- and class II-knockout NOD mice fail to develop diabetes (6, 7). The activation of autoreactive T-cells in diabetes requires the recognition of auto-antigens expressed by β-cells as well as costimulatory signals (8). Membrane proteins of the *Cd28* gene family mediate costimulatory signals through their interaction with members of the B7 family expressed on antigen-presenting cells and stromal cells (9). *In vivo* deletion of the *Cd28* or B*7* genes and *Icos* or *Icosl* genes profoundly affects the development of diabetes in the NOD mouse by modulating effector and/or regulatory T-cells (10–14).

NOD mice, as humans, are susceptible to the development of other forms of autoimmunity and occasionally develop infiltrates in the thyroid, the parathyroid, the adrenal, and salivary glands (4, 15–17). This predisposition to autoimmunity also sets up NOD mice as a relevant model for experimental induction of autoimmune diseases such as autoimmune prostatitis or autoimmune thyroiditis (16, 18, 19). Different NOD genes are involved in orienting the autoimmune response toward β-cells. The main region controlling the targeting of β-cells is the MHC, but other regions have been evidenced in double congenic mice (20–24). Finally, genes controling costimulatory T-cells molecules have been shown to play a key role in directing autoimmunity, as observed in B7.2-knockout NOD mice, which fail to develop diabetes but develop autoimmune peripheral neuropathy (25, 26).

We previously reported that protection from diabetes in ICOS^−/−^ NOD mice was unexpectedly associated with the development of an autoimmune disorder of the neuro-muscular system, characterized by myositis and sensory ganglionitis. In this model, defective activation of diabetogenic effector ICOS^−/−^T-cells and a defect in Treg cells result in the protection of ICOS^−/−^ NOD mice from diabetes (14). Here, we focused on ICOSL^−/−^ NOD mice which, in contrast to ICOS^−/−^ NOD mice, only carry in their genome a limited C57BL/6 region containing the *Icosl* nul mutation but no gene variant previously reported as associated to NOD diabetes. This study describes the autoimmune neuromyopathy that predominantly occurs in females and manifests clinically by locomotor disability first affecting the front paws. We show that neuromuscular autoimmunity is associated to a CD4^+^ Th1 profile, fails to develop in mice lacking CD4^+^ but not CD8^+^ T-cells, and is transferable by CD4^+^ T-cells. This definitively demonstrates the autoimmune character and MHC class II restriction of the neuromyopathy.

## Animals and Methods

### Mice

NOD mice were bred and housed in our facilities under specific pathogen-free conditions. ICOS^−/−^ and ICOSL^−/−^ NOD mice were generated as described previously (14).

ICOS^−/−^ICOSL^−/−^ NOD mice were established by crossing ICOS^−/−^ NOD with ICOSL^−/−^ NOD mice to generate F1 mice, and F1 mice were repeatedly intercrossed together to produce homozygous mice.

CIITA^−/−^ICOSL^−/−^ and β2microglobulin (β2m)^−/−^ICOSL^−/−^ NOD mice were established similarly by crossing ICOSL^−/−^ NOD mice with CIITA^−/−^ NOD mice and β2m^−/−^ NOD mice (Jackson laboratory, Bar Harbor, ME, USA), respectively. ICOSL^−/−^ NOD.*scid* mice were obtained by crossing NOD.*scid* mice with ICOSL^−/−^ NOD mice. The prevalence of diabetes in our NOD colony reaches 10% in males and 60% in females by 6 months of age. Animal studies were approved by institutional review.

### Genetic Analysis

Genomic DNA extracted from mouse tail tips using standard protocols was processed and hybridized on Affymetrix Mouse Diversity Genotyping Arrays (Santa Clara, CA, USA) according to the manufacturer’s instructions. For data extraction, genome coordinates were determined using the assemblies UCSC version mm10 and NCBI version GRCm38. Non-informative markers were manually removed for further comparative strain analysis. In all, 56,690 markers were evaluated for the ICOS^−/−^ NOD mice, 558,318 for the ICOSL^−/−^ NOD mice when compared to NOD mice.

### Diabetes and Neuromyopathy Assessments

Diabetes was assessed by monitoring mice as described previously (14). Neuromyopathy was quantified using a clinical score as follows. Stage 1 is defined by asymmetric extension of back legs after tail-suspension test. Stage 2 is defined by retraction of back legs after tail-suspension test. Stage 3 is defined by flexion of front and back legs without snatching to the grid after tail-suspension test.

Weight variations were monitored longitudinally, and muscle strength was assessed by grip test performance (Bioseb) on the front paws. Locomotor activity was analyzed using a Catwalk apparatus (Noldus) comprising a high-speed color camera for accurate spatial and temporal resolution, real-time movement recording, and automated analysis of the locomotor ability.

### Histological Analysis

Gastrocnemius were dissected and frozen in cold isopentane. Sections of 4 μm from these muscles were then stained with hematoxylin and eosin. Immunohistochemistry was performed on frozen muscle sections using the Vectastain elite ABC peroxidase kit (Vector). Sections were stained with primary Abs directed against CD4 (GK1.5), CD8 (53-6.7), F4/80 (BM8) from eBioscience, CD11b (M1/70) from BD Pharmingen, or MYH3 (F1.652) from Santa Cruz Biotechnology, then revealed with an appropriate secondary antibody if necessary and streptavidin-peroxydase using amino-ethyl-carbazole (Vector) as substrate.

Alternatively, anesthetized animals were perfused with a 4% formaldehyde solution (Electron Microscopy Science, Hatfield, PA, USA), then brain, spinal cord, spinal roots, spinal and trigeminal ganglia, and pieces of different skeletal muscles were dissected and embedded into paraffin. Three sections were stained with hematoxylin and eosin. Immune infiltration was estimated and graded from 0 to 3. Immunocytochemistry was performed on frozen muscle preparations. Sections were stained with primary antibody specific to: CD8 (53-6.7), CD4 (RM4-5), I-Ad (AMS-32.1), H-2Kd (SF1-1.1), CD11b (M1/70), or CD45R/B220 (RA3-6B2) from BD Bioscience. Histologic sections were labeled with a secondary antibody (Anti-rat biotin from eBioscience and/or streptavidine-594 from Invitrogen), and cell nuclei were counterstained using Hoechst.

### Antibody Isotype Quantification

Maxisorp plates were coated using one of the following Abs: anti-IgG1, anti-IgG2a, anti-IgG2b, anti-IgG3, anti-IgM (Southern Biotech). After incubation with mouse sera, revelation was performed using biotin-coupled anti-mouse immunoglobulin (Ig) (H + L) (Jackson IR), streptavidin HRP (ThermoFisher), TMB, and H_2_SO_4_. Optical densities were assessed using a ThermoScientific Multiskan FC apparatus.

### Flow Cytometry Analysis

#### Antibodies

The following Abs were used purified or conjugated to biotin (hybridoma clone indicated in parentheses): CD4 (RM4-5), CD8 (53-6.7), B220 (RA3-6B2), CD11b (ICRF44), CD11c from BD Bioscience and IFN-γ (4S.B3), IL-4 (B-S4), IL-17 (17B7), FOXP3 (FJK-16s), and ICOSL (HK5.3) from eBioscience. Appropriate isotype control mAbs were included. Living cells were gated on side scatter *versus* forward scatter density plot.

#### Immunofluorescence Staining

Immunofluorescence staining was performed as described previously (14). Intracellular cytokine staining was performed according to the manufacturer’s instructions (staining intracellular antigens; eBioscience). Flow cytometryc analyses were performed with a FACS FORTESSA (Becton Dickinson), using the FlowJo software (Tree Star, Ashland, OR, USA).

#### Cytokine Production

5 × 10^4^ CD4^+^ T-cells from 8-week-old ICOSL^+/+^ or ICOSL^−/−^ NOD mice were cultured in triplicate in 96-well plates with 1 × 10^5^ irradiated NOD splenocytes, phorbol 12 myristate 13-acetate (PMA 10 ng/ml; Sigma), ionomycin (1 μg/ml; Sigma), and brefeldin A (10 μg/ml; Sigma). CD4^+^ T-cells were incubated 4 h at 37°C. Cells were collected and tested for IFN-γ, IL-4, and IL-17 production by flow cytometry gated on CD4^+^ T-cells.

### Immunomagnetic Cell Sorting

CD4^+^ T-cells were obtained using mouse CD4^−^ Negative Selection Kit (Invitrogen Dynal AS, Carlsbad, CA, USA), according to the manufacturer’s instructions. CD8^+^ T-cells/CD4^+^ and CD8^+^ T-cells/CD4^+^CD25^+^ T-cells or CD4^+^CD25^−^ T-cells were positively selected by MACS sorting with biotinylated anti-CD4 and/or anti-CD8 Abs, biotin-anti-CD25 (BD Bioscience) Abs using LS or LD columns and streptavidin beads according to the manufacturer’s instructions (Miltenyi Biotec, Bergisch Gladbach, Germany).

### Adoptive Cell Transfer

Purified CD4^+^ T-cells, CD8^+^ T-cells, T-cells including CD4^+^ T-cells and CD8^+^ T-cells, splenocytes depleted of CD4^+^ and CD8^+^ T-cells from 8 or 40-week-old either ICOSL^+/+^ or ICOSL^−/−^ NOD mice were selected as described above. When indicated, cells were then stimulated with 1 μg/ml anti-CD3 (BD Bioscience) and anti-CD28 (BD Bioscience) for 48 h. IL-2 (20 U/ml, R&D Systems, Minneapolis, MN, USA) was added to the culture of the splenocytes depleted of CD4^+^ and CD8^+^ T-cells. Total spleen (SPL) cells or purified T-cells, stimulated or not, were then adoptively transferred i.v. into 6- to 8-week-old ICOSL^+/+^ or ICOSL^−/−^ NOD.*scid* mice as previously described (14). Ten days after transfer and up to 15 weeks post-transfer, mice were tested twice a week for glycosuria and neuromyopathy scored. Anesthetized mice were then perfused intracardially by 4% formaldehyde solution, in order to perform histological analysis (Electron Microscopy Science, Hatfield, PA, USA).

### Quantitative RT-PCR Experiments

Total RNA from cultured mouse brain, spinal cord, muscle, lymphoid cells, and Schwann cell line (MSC80) were obtained using TRIzol^®^ Reagent (Invitrogen, France). One microgram of total RNA was reverse transcribed with random primers from Promega (Charbonnières, France) and reverse transcriptase M-MLV-RT from Invitrogen (Cergy Pontoise, France).

Quantitative real-time PCR was performed with standard protocols using SYBR^®^Green ROX Mix (Thermo Scientific, France) as a fluorescent detection dye in ABI PRISM^®^ 7000 in a final volume of 10 μl which also contains 300 nM primers (Operon, Germany) and 20 ng of reverse transcribed RNA in 384-well plates. To characterize the generated amplicons and to control the contamination by unspecific by-products, a melting curve analysis was employed. Each reaction was performed in triplicate, and the mean of at least three independent experiments was calculated. All results were normalized to the 26S mRNA level and calculated using the Delta Ct method. The primer sequences used in real-time qPCR are:

iCOS F = TAGGGTGTGCAGCTTTCGTT
iCOS R = AGCTTATGAGGTCACACCTGC
iCOSL F = CAGCGGCATTCGTTTCCTTC
iCOSL R = GTCAGGCGTGGTCTGTAAGT

### Statistical Analysis

Appropriate statistical tests (log rank or student *t* test) were performed as indicated in figure legends using GraphPad Prism Version 4.0b software (GraphPad Software, La Jolla, CA, USA).

### Ethics Approval

This study was carried out in accordance to the recommendations of Institutional Animal Care and Use Guidelines. The protocol was approved by the ethic committee under number CEEA34.CB.024.11.

## Results

### Genetic Characterization of ICOS^−/−^ and ICOSL^−/−^ NOD Mice

To study the role of the ICOS costimulation pathway in NOD mice, we initially used ICOS^−/−^ or ICOSL^−/−^ animals that were backcrossed onto a NOD genetic background. In ICOS^−/−^ NOD mice, we detected a large interval of 39.6 Mb between the markers rs31808238 and rs36471198 almost entirely composed of SNPs of non-NOD origin (data not shown). This interval overlaps with the *Idd* regions 5.1, 5.2, and 5.3 (Table 1). However, *Idd5* has been experimentally excluded to have any effect on neuropathy (26).

**Table 1:** **Size of the non-NOD-derived genomic regions around the mutated genes expressing ICOS and ICOS/ICOS ligand in non-obese diabetic congenic mice, and comparison of these intervals to known candidate regions for type 1 diabetes (*Idd* loci) and neuropathy (QTLs)**.

	Base pairs		*Idd*/marker	
**Intervals of the *Idds***				
chr1	60,826,239	62,793,632	*Idd5.1*	
	66,483,902	70,037,817	*Idd5.3*	
	73,937,554	75,468,438	*Idd5.2*	
**Non-NOD interval of the *Icos*^−/−^ knockout**				
chr1	3,544,819	7,501,347	rs31808238	rs36471198
**non-NOD SNPs containing intervals in the *Icosl*^−/−^ knockout**				
chr 10	74,298,599	75,507,309	rs51825371	rs13471390
	84,580,657	85,644,817	rs29371540	rs51263527
	86,197,418	86,437,612	rs30182742	rs30196734

In the ICOSL^−/−^ strain, we detected an interval of 1.2 Mb around the *Icosl* gene (rs51825371 to rs13471390), that was less than half, but not entirely, composed of non-NOD alleles (data not shown). In addition, two distal intervals of 1.1 Mb and 240 kb with similar composition were detected between the markers rs29371540 and rs51263527, and rs30182742 and rs30196734, respectively (Table 1). All three intervals do not overlap with any known *Idd* intervals. They do also not correspond to previous QTL locations for neuropathy on chromosome 10, e.g., *Annp9* that has been mapped further proximal at the marker rs13480629 (chr 10; 67,011,671 bp) (27). Therefore, we chose to study ICOSL^−/−^ rather than ICOS^−/−^ NOD mice in this report.

### ICOS^−/−^, ICOSL^−/−^, and ICOS^−/−^ICOSL^−/−^ Double-Knockout NOD Mice Are Protected from T1D and Develop Neuromuscular Autoimmunity

**Figure 1:**
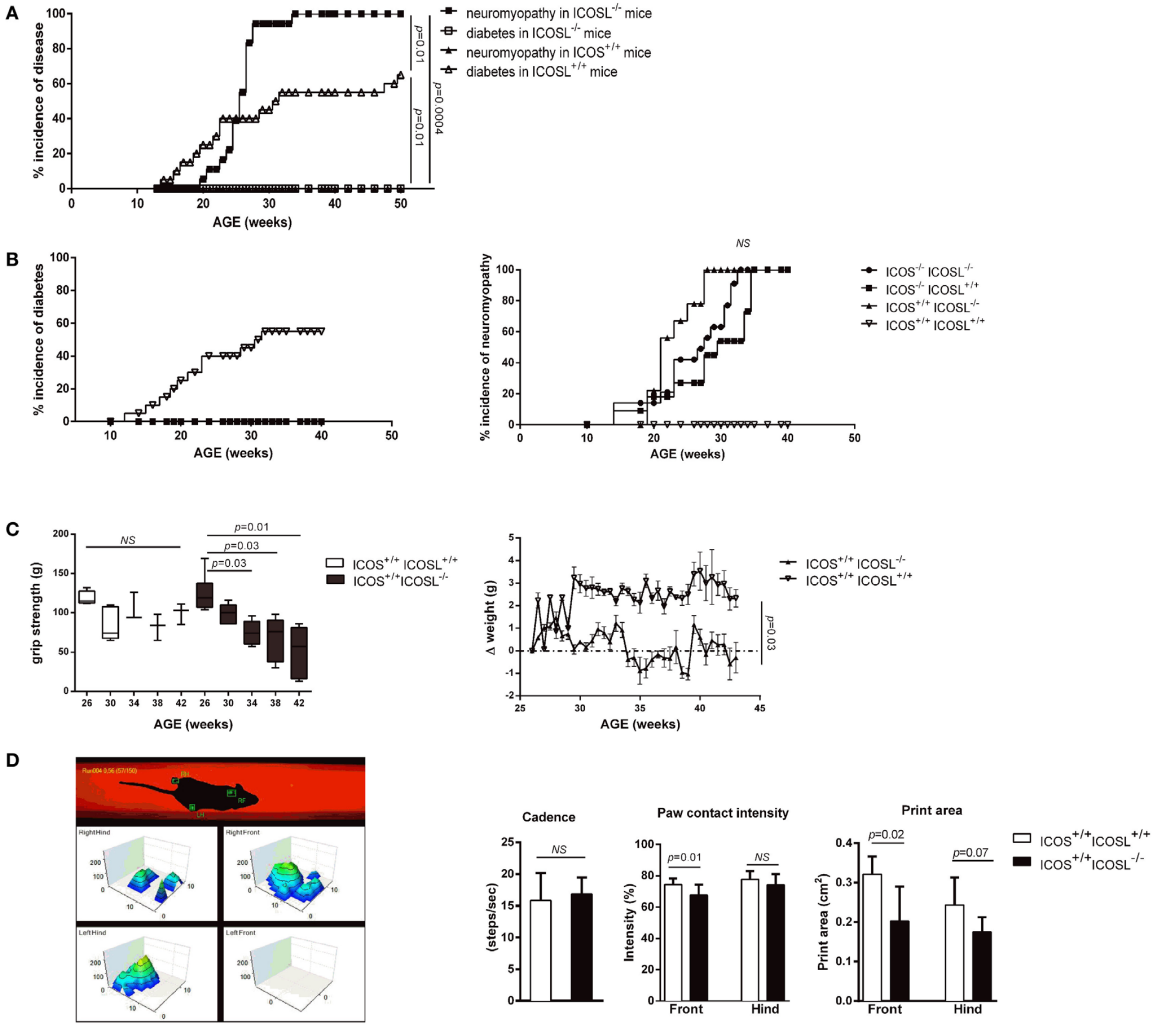

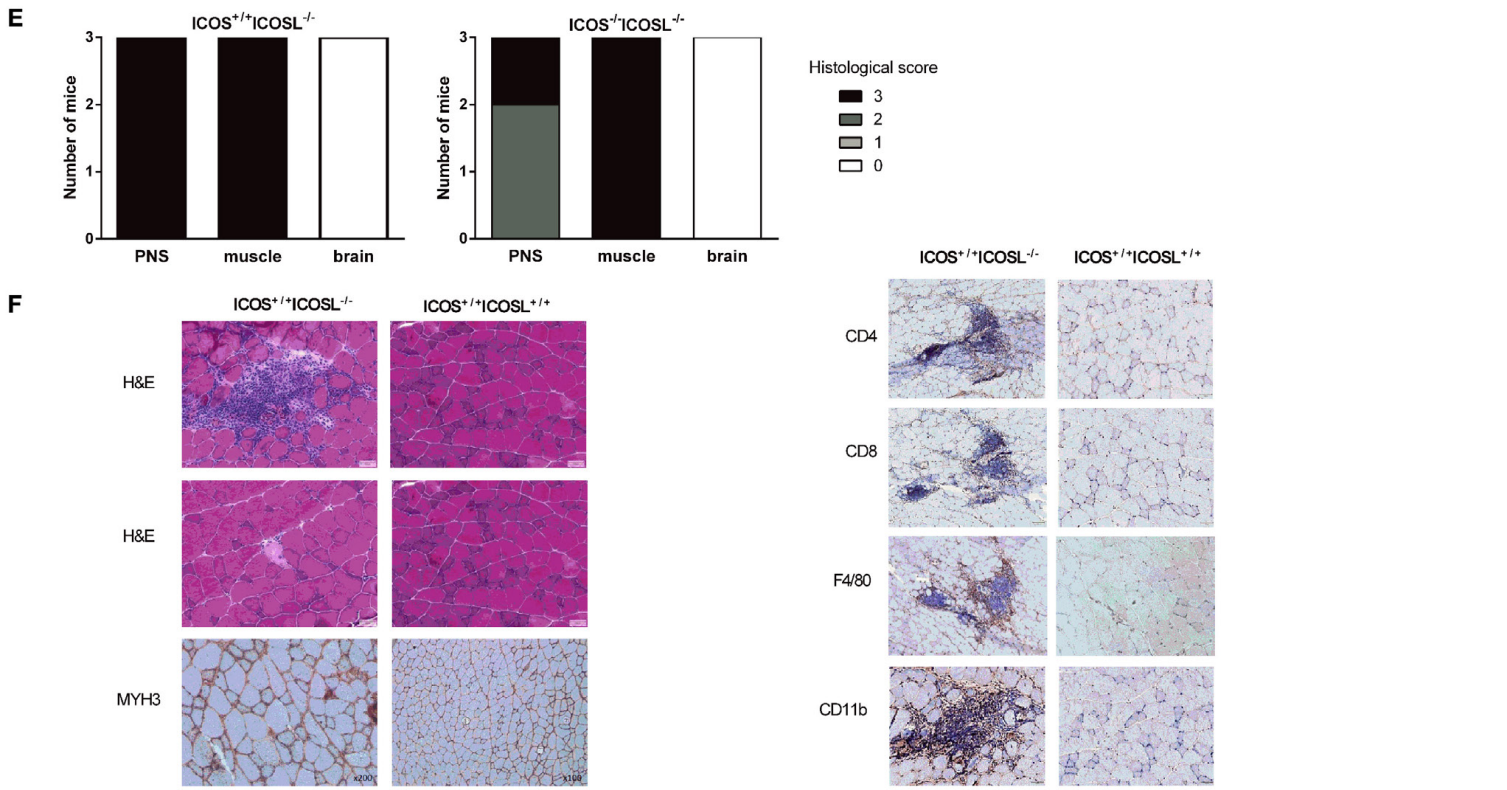
**ICOS^−/−^, ICOSL^−/−^, and ICOS^−/−^ICOSL^−/−^ double-knockout non-obese diabetic (NOD) mice are protected from T1D and develop neuromuscular autoimmunity**. **(A)** Incidence of clinical neuromyopathy (■) and diabetes (□) in female ICOSL^−/−^ NOD mice (*n* = 18 mice) and incidence of neuromyopathy (▴) and diabetes (Δ) in female ICOSL^+/+^ NOD mice (*n* = 20 mice) (Log-Rank Test). **(B)** Spontaneous Incidence of clinical diabetes and neuromyopathy in female ICOS^−/−^ ICOSL^+/+^ (■, *n* = 11), ICOS^+/+^ICOSL^−/−^ (▴, *n* = 10), ICOS^−/−^ICOSL^−/−^ (●, *n* = 13), and ICOS^+/+^ICOSL^+/+^ NOD mice (▿, *n* = 14) (Log-Rank Test). **(C)** Functional impairment in ICOSL^−/−^ NOD mice. Muscle strength assessed by grip test performance in ICOS^+/+^ICOSL^−/−^ (black, *n* = 6) and ICOS^+/+^ICOSL^+/+^ NOD mice (white, *n* = 5) (Student *t*-test). Weight variations in female ICOS^+/+^ICOSL^−/−^ (▴, *n* = 5) and ICOS^+/+^ICOSL^+/+^ NOD mice (▿, *n* = 3). **(D)** Locomotor activity assessed by catwalk analysis. Cadence (steps/s), print area (cm^2^), and paw contact intensity (%) were assessed in 35-week-old female ICOS^+/+^ICOSL^−/−^ (black, *n* = 10) and ICOS^+/+^ICOSL^+/+^ NOD mice (white, *n* = 15) (Student *t*-test). **(E)** Histologic score of the cell infiltrate in peripheral nerve system, muscle, and brain in female ICOS^+/+^ICOSL^−/−^ and ICOS^−/−^ICOSL^−/−^ NOD mice. **(F)** Representative sections of gastrocnemius from 34-week-old female ICOSL^−/−^ and ICOS^+/+^ICOSL^+/+^ NOD mice. Hematoxylin/eosin (H&E) stainings showing an endomysial mononuclear infiltrate and necrotic myofibers. MYH3 immunostainings highlight regenerating myofibers, and immunostaining for CD4^+^, CD8^+^ T-cells, F4/80^+^ macrophages, and CD11b^+^ myeloid cells within infiltrates.

As we previously reported, both ICOS^−/−^ and ICOSL^−/−^ NOD mice are protected from diabetes but develop a severe neuromyopathy (14). As shown in Figure 1A, diabetes developed from the age of 12 weeks in female wild-type (WT) ICOSL^+/+^ NOD mice and reached a plateau at around 32 weeks of age with a final prevalence of diabetes of 65% at 50 weeks. At 24 weeks of age, ICOSL^−/−^ NOD mice remained free of insulitis with very few lymphocytes seen in the periphery of the islets, in contrast with ICOS^−/−^ NOD mice in which a peri-insulitis was still observed (data not shown). Female ICOSL^−/−^ NOD mice developed clinical neuromuscular disease from 19 weeks to reach a plateau around 34 weeks of age. Overall, the timescale of neuromyopathy onset paralleled that of diabetes with a 6–8 weeks delay (*p* = 0.01; Log-Rank Test). However, if the prevalence of diabetes reached 65% by 50 weeks of age in ICOSL^+/+^ NOD female mice, the prevalence of neuromyopathy was 100% in female ICOSL^−/−^ NOD mice (Figure 1A) and 60% in male ICOSL^−/−^ (not shown) by 50 weeks of age. We then compared female ICOS^−/−^ and ICOSL^−/−^ NOD mice and addressed whether the incidence was comparable in ICOS^−/−^ICOSL^−/−^ double-knockout mice. ICOS^−/−^, ICOSL^−/−^, and ICOS^−/−^ICOSL^−/−^ NOD mice were all protected from diabetes and developed neuromyopathy following similar survival curves, but median age of neuropathy was younger in ICOSL^−/−^ (22 weeks) versus ICOS^−/−^ NOD mice (30 weeks, *p* = 0.008) (Figure 1B). ICOS^+/+^ICOSL^−/−^ NOD mice presented a significant loss of grip strength at 34 weeks of age as compared to ICOS^+/+^ICOSL^+/+^ NOD mice (Figure 1C) and a significant loss of weight at 43 weeks of age (Figure 1C). Locomotor disability first affected the front paws in ICOS^+/+^ICOSL^−/−^ NOD mice, as manifest by reduction in print area and paw contact intensity (Figure 1D). Heterozygous ICOSL^±^ NOD females maintained a high prevalence of diabetes (71%, *n* = 20/28) and, when remaining diabetes-free, developed neuromyopathy (87%, *n* = 7/8 at 30 weeks) (data not shown).

On histological sections, muscle and nerve infiltrates were already present in 24-week-old female mice but were not detected in 16-week-old female mice (data not shown). The severity of the neuromuscular infiltrate was identical in female ICOS^+/+^ICOSL^−/−^ and ICOS^−/−^ICOSL^−/−^ NOD mice (Figure 1E). High grade immune infiltration was detected in muscles and peripheral nerves of 40-week-old female ICOS^+/+^ICOSL^−/−^ NOD mice (Figure 1F) but also in male ICOS^+/+^ICOSL^−/−^ NOD mice despite the lower prevalence of clinical neuromuscular disease (data not shown). Muscular infiltration was associated with evidence of necrosis and regeneration and composed of CD4^+^ T-cells, CD8^+^ T-cells, F4/80^+^ macrophages, and CD11b^+^ myeloid cells (Figure 1F). At 24 weeks of age, it was possible to recover infiltrating cells from muscles of ICOSL^−/−^ NOD mice and to analyze by flow cytometry the composition of the infiltrate: 31% B220^+^ cells, 26% CD8^+^ T-cells, and 54% CD4^+^ T-cells among TCR^+^T-cells, with 7% of CD4^+^Foxp3^+^ T-cells among CD4^+^ T-cells (data not shown).

### Immune Phenotype of ICOSL^−/−^ NOD Mice

**Figure 2:**
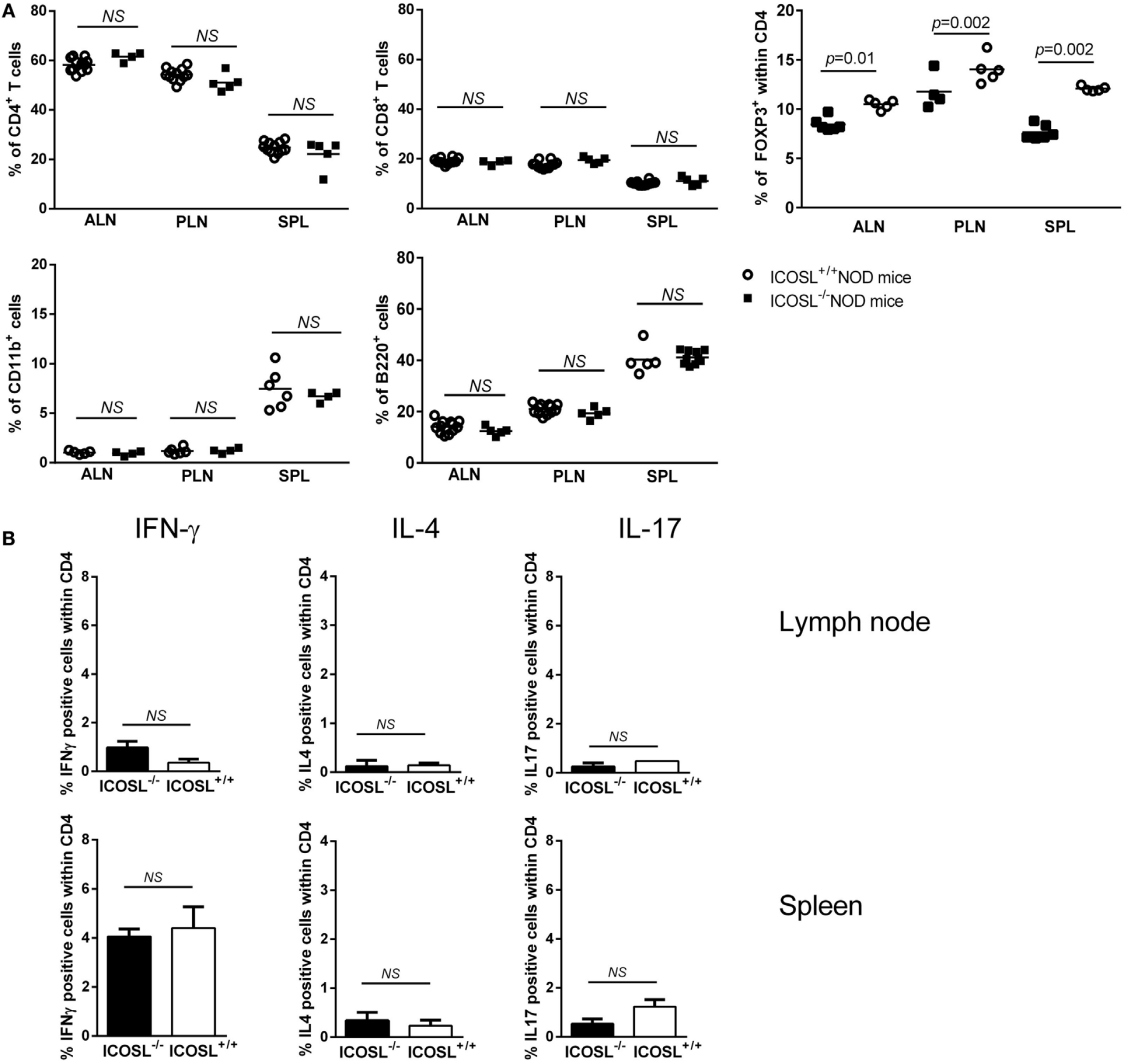

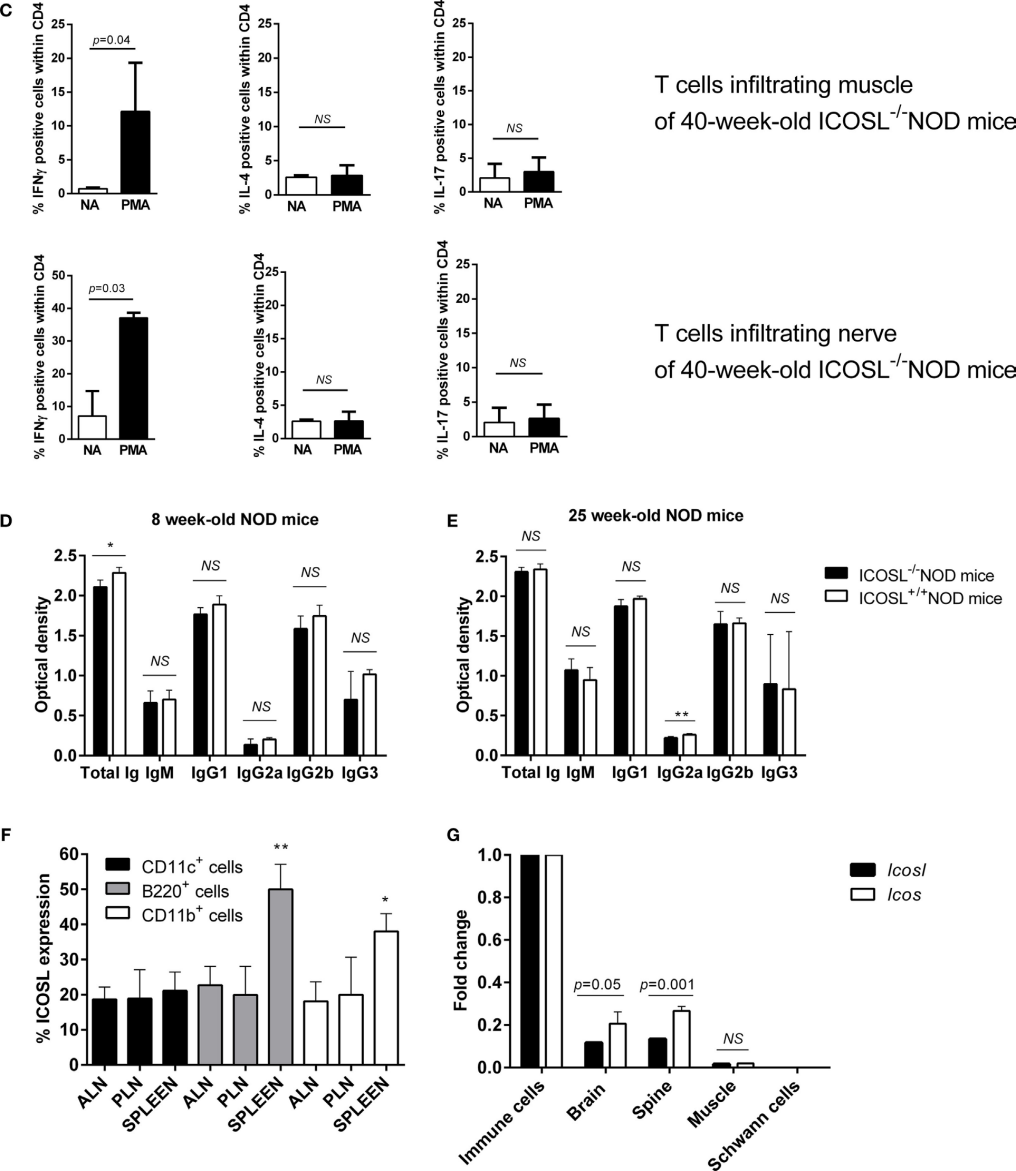
**Immune phenotype of ICOSL^−/−^ non-obese diabetic (NOD) mice**. **(A)** Percentage of CD4^+^, CD8^+^, B220^+^, CD11b^+^, and CD4^+^FOXP3^+^ cells in spleen (SPL), axillary lymph node (ALN), pancreatic lymph node (PLN), and SPL of ICOSL^+/+^ and ICOSL^−/−^ NOD mice at 6-week-old (*n* = 6, NS: *p* > 0.05, Mann–Whitney test). **(B)** IFN-γ, IL-4, and IL-17 cytokine production by CD4^+^ T-cells from lymph node, SPL after PMA iomonycine stimulation. **(C)** IFN-γ, IL-4, and IL-17 cytokine production by CD4^+^ T-cells infiltrating muscle and nerves of ICOSL^−/−^ NOD mice after PMA iomonycine stimulation (NA, non-activated). Data are representative of three independent experiments. Immunoglobin isotypes levels in sera from 8-week-old mice, before neuromuscular disease onset **(D)** and from 25-week-old mice **(E)** in ICOSL^−/−^ and ICOSL^+/+^ NOD mice (*n* = 5, NS: *p* > 0.05, *p < 0.05 **p < 0.005, Mann–Whitney test). **(F)** Expression of ICOS/ICOS ligand (ICOSL) on B220^+^, CD11c^+^, and CD11b^+^ cells in ALN, PLN, and SPL of 6-week-old ICOSL^+/+^ NOD mice (*n* = 6, *p < 0.05, **p < 0.005, Mann–Whitney test). **(G)** *Icos* and *Icosl* expression on brain, Schwann cells, spine, and muscle of control mice (Mann–Whitney test).

We further studied the CD11b^+^ cells, CD4^+^ T-cells, CD8^+^ T-cells, and B220^+^ cells in axillary lymph node (ALN), pancreatic lymph node (PLN), and SPL in ICOSL^−/−^ NOD mice as compared to WT NOD mice at 6 weeks of age. These populations were comparable in ICOSL^−/−^ NOD mice and in WT NOD mice (Figure 2A). Interestingly, a reduction of 25–32% FOXP3^+^CD25^+^CD4^+^ T-cells was seen in ICOSL^−/−^ mice as compared to WT mice, respectively, in PLN and SPL (Figure 2A). Cytokine production by CD4^+^ T-cells was analyzed by flow cytometry following PMA-ionomycin activation. The IFN-γ, IL-4, and IL-17 production were not different in ICOSL^−/−^ NOD mice and ICOSL^+/+^ NOD mice in SPL or lymph node (Figure 2B). But strikingly muscle and nerves infiltrating T-cells from 40-week-old ICOSL^−/−^ NOD mice produced INF-γ but not IL-4 or IL-17 after PMA-ionomycin stimulation (Figure 2C).

Because ICOS is expressed by follicular helper T-cells that are involved in the class switch from IgM to IgG subclasses, we investigated whether serum Ig isotypes could be affected in ICOSL^−/−^ NOD mice. Before neuromuscular disease onset (8 weeks), we only observed a slight decrease in total Ig amount in ICOSL^−/−^ NOD mice sera as compared to ICOSL^+/+^ NOD mice (Figure 2D). Yet, there was no difference in Ig subclasses between ICOSL^−/−^ NOD and ICOSL^+/+^ NOD mice. Also, when disease was apparent at 25 weeks, no difference between the ICOSL^−/−^ and ICOSL^+/+^ NOD mice was either observed, except a minor variation in IgG2a levels (Figure 2E). Therefore, the neuromyopathy developed by ICOSL^−/−^ NOD mice is very unlikely to be linked to a default in Ig switch.

Because ICOSL is constitutively expressed on antigen-presenting cells, we studied its expression in B-cells, dendritic cells, and myeloid cells of our mice. ICOSL was present at the surface of about 20% of B-cells, myeloid, and dendritic cells in pancreatic and ALN cells of 6-week-old ICOSL^+/+^ NOD mice. In the SPL, ICOSL was expressed on almost 50% of B-cells, 40% of myeloid cells, and 21% of dendritic cells (Figure 2F). Regarding mRNA expression, *Icos* and *Icosl*, were expressed in brain, in spine but not Schwann cells of control mice. In muscle, mRNA expression was found very low (Figure 2G).

### The Neuromyopathy Is a CD4^+^ T Cell-Dependent Autoimmune Disease

**Figure 3:**
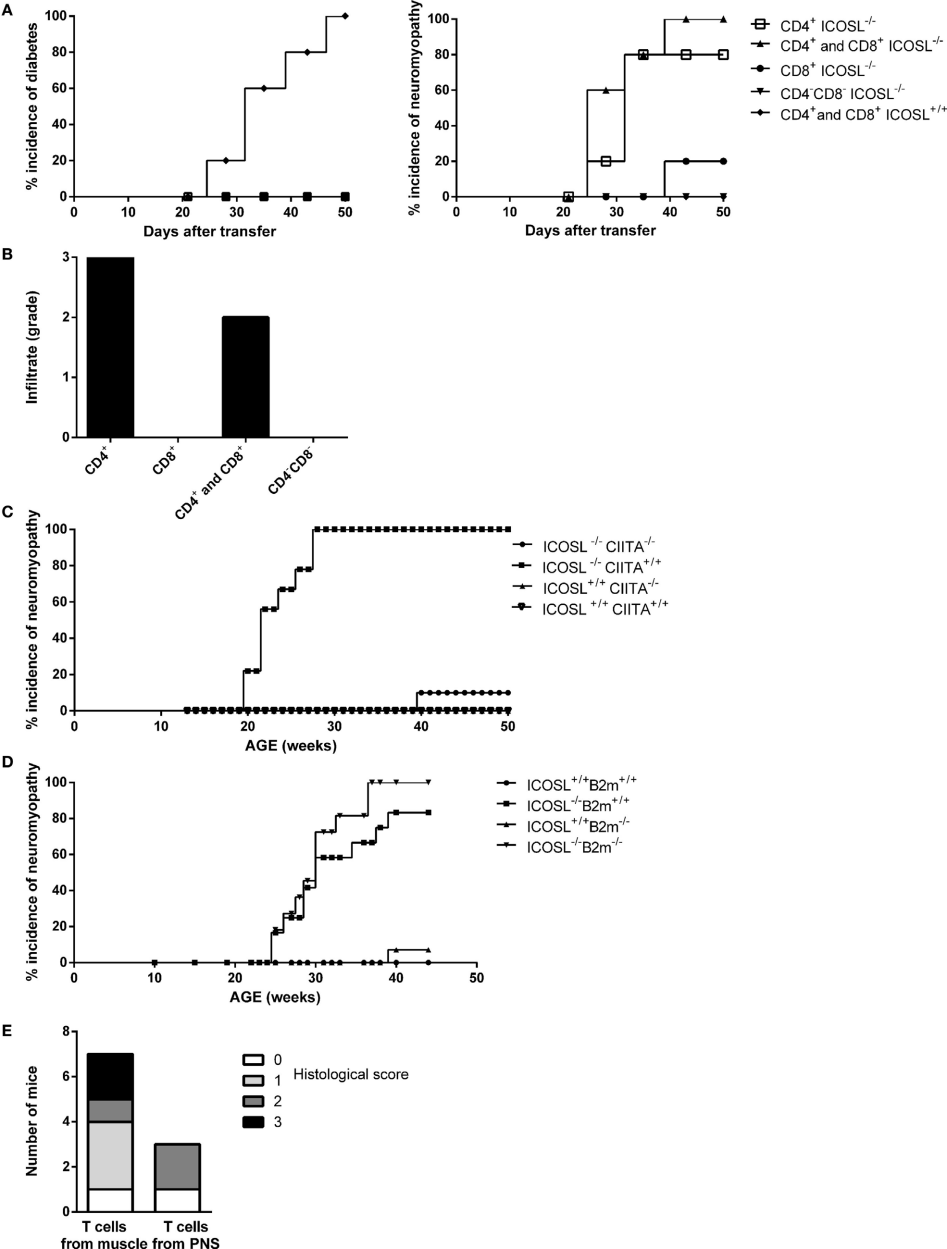
**Neuromyopathy is CD4^+^ T cell-dependent**. **(A)** Transfert of spleen (SPL) T-cells from ICOSL^−/−^ old mice into non-obese diabetic (NOD).*scid* mice. Eight-week-old female NOD.*scid* recipient mice were transferred with 0.8 × 10^6^ stimulated CD4^+^ T-cells (■), 0.8 × 10^6^ stimulated CD8^+^ T-cells (ν), 0.8 × 10^6^ purified stimulated T-cells including CD4^+^ and CD8^+^ T-cells (▴), or 0.8 × 10^6^ stimulated splenocyte depleted of CD4^+^ and CD8^+^ T-cells called “CD4^−^ CD8^−^” (▾) from 40-week-old ICOSL^−/−^ NOD mice (*n* = 6, data are representative of three independent experiments). **(B)** Histological scores of infiltrating cells in muscle and peripheral nervous system of transferred recipients. **(C)** Incidence of neuromyopathy in ICOSL^−/−^CIITA^−/−^ NOD mice. **(D)** Incidence of neuromyopathy in ICOSL^−/−^beta2m^−/−^ NOD mice. **(E)** Histological scores of infiltrating cells in muscle and peripheral nervous system of recipient mice after transfer of T-cells isolated from muscles (10^6^ cells) and nerves (0.2 × 10^6^ cells) of 40-week-old ICOSL^−/−^ NOD mice.

In order to address whether the neuromuscular syndrome observed in ICOSL^−/−^ NOD mice was dependent on both CD4^+^ and CD8^+^ T-cells, as seen for diabetes, or on either of these two subsets, we performed adoptive transfer experiments. When CD4^+^ T-cells were transferred in association with CD8^+^ T-cells from diseased ICOSL^−/−^ NOD mice or even as a single subset into NOD.*scid* recipient mice, both recipient groups developed neuromyopathy but not diabetes. Development of clinical neuromyopathy was observed as early as by 25 days post-transfer and in 80–100% recipients within 50 days post-transfer. By contrast, ICOSL^−/−^ CD8^+^ T-cells alone or splenocytes depleted in CD4^+^ T-cells and CD8^+^ T-cells (CD4^−^CD8^−^ T-cells) did transfer neither neuromyopathy nor diabetes (Figure 3A). Histological analyses of recipient mice confirmed that either CD4^+^ T-cells or CD4^+^ T-cells in association with CD8^+^ T-cells transfer a neuromuscular infiltrate in NOD.*scid* recipients (Figure 3B). The composition of the infiltrate observed in muscles and nerves was identical to that observed in the spontaneously diseased mice (data not shown).

To confirm that autoimmune neuromyopathy development in ICOSL^−/−^ NOD mice was CD4^+^ T cell-dependent, we studied ICOSL^−/−^ NOD mice that were further deficient in either the *C2ta* or the β*2-m* genes. These mice were deprived of either class II-restricted CD4^+^ T-cells or class I-restricted CD8^+^ T-cells, respectively. ICOSL^−/−^CIITA^−/−^ NOD mice did not develop autoimmune neuromyopathy, in contrast to control ICOSL^−/−^CIITA^+/+^ NOD mice (Figure 3C). They did not develop diabetes over 40 weeks of survey (data not shown). By contrast, ICOSL^−/−^β2-m^−/−^ NOD mice developed a neuromyopathy, along with a neuromuscular infiltrate that was comparable to that developed by ICOSL^−/−^ β2m^+/+^ control NOD mice (Figure 3D). Thus, both ICOSL^−/−^CIITA^−/−^ and ICOSL^−/−^β2m^−/−^ NOD mice were protected from diabetes, but only ICOSL^−/−^CIITA^−/−^ NOD mice were protected from neuromuscular autoimmunity, confirming the major pathogenic role of CD4^+^ T-cells in the neuromuscular model.

We further investigated whether muscle- and nerve-infiltrating cells were able to directly transfer nerve and/or muscle autoimmunity. We isolated muscle and nerve infiltrates and transferred them into NOD.*scid* mice. Recipients developed clinical neuromyopathy following the transfer of either nerve (two recipient mice out of three) or muscle (six recipient mice out of seven) infiltrating T-cells. Nerve-infiltrating T-cells induced nerve and muscular infiltrates in NOD.*scid* recipients, although, the number of infiltrating cells recovered from nerve, and transferred (0.2 × 10^6^) was fivefold lower as compared to cells recovered from infiltrated muscle and transferred into NOD.*scid* recipients (1.0 × 10^6^) (Figure 3E).

### ICOS/ICOSL Is Not Required for Efficient Transfer of Neuromyopathy

**Figure 4:**
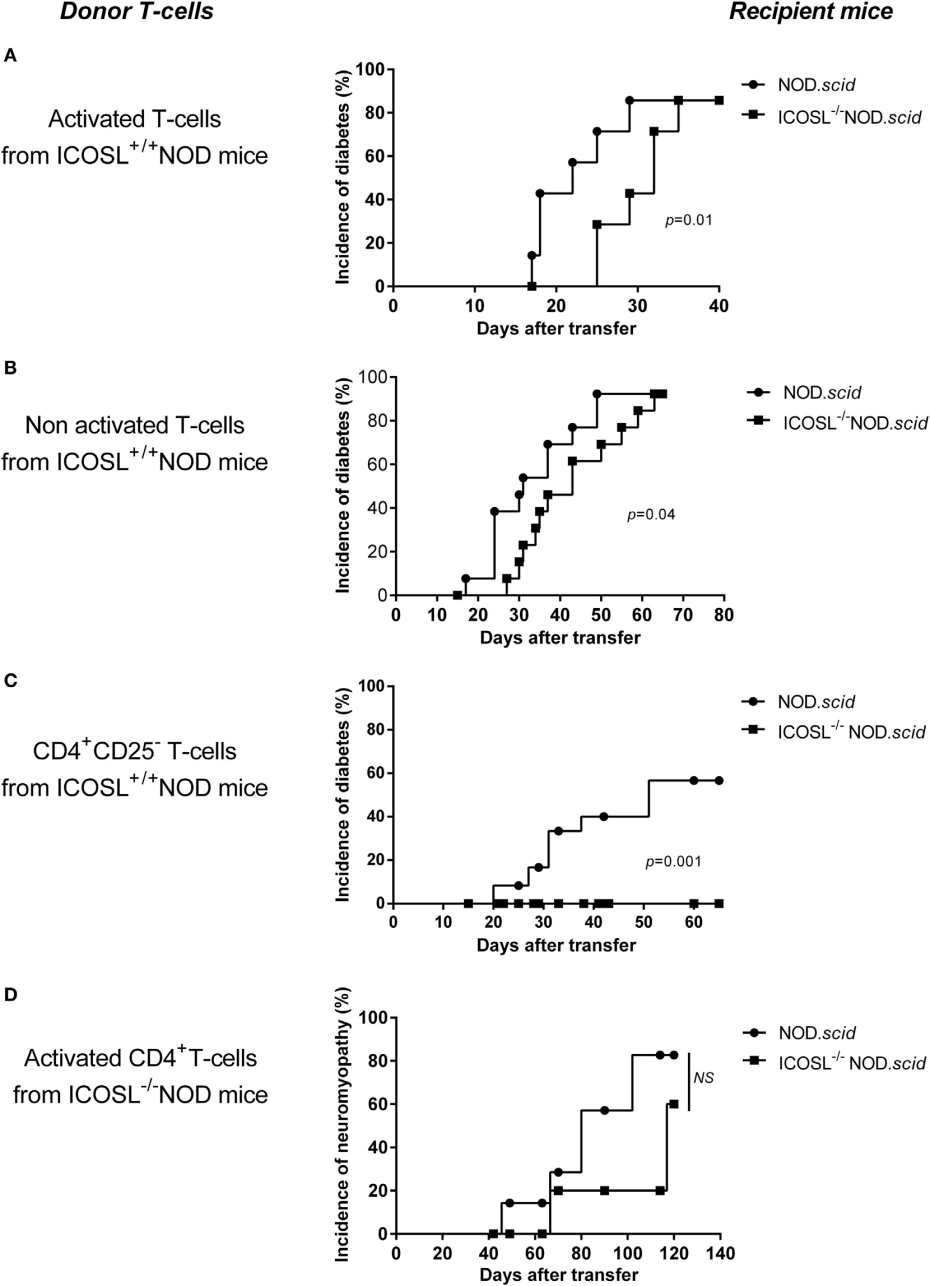
**ICOS/ICOSL signal is not required for efficient transfer of neuromyopathy**. **(A–C)** Eight-week-old recipient ICOSL^+/+^ (●, *n* = 8) or ICOSL^−/−^ NOD.*scid* mice (■, *n* = 8) were transferred with 6 × 10^6^ purified SPL cells including CD4^+^ and CD8^+^ T-cells from 40-week-old ICOSL^+/+^ NOD mice activated *in vitro* with anti-CD28 and anti-CD3 antibody **(A)**, or with 6 × 10^6^ unactivated purified SPL cells including CD4^+^ and CD8^+^ T-cells from 40-week-old ICOSL^+/+^ NOD mice **(B)**, or with 6 × 10^6^ unactivated CD4^+^CD25^−^ T-cells from 40-week-old ICOSL^+/+^ NOD mice **(C)**. In **(D)**, ICOSL^+/+^ (●, *n* = 10) or ICOSL^−/−^ (■, *n* = 10) female NOD.*scid* were transferred with 1 × 10^6^ activated CD4^+^ T-cells from 40-week-old ICOSL^−/−^ NOD mice (Log-Rank Test).

The ICOS/ICOSL pathway has been shown central in the primary activation of naive T-cells (28). Whether it is also required for activation of autoreactive effector cells remains an open issue. CD8^+^ T-cells and CD4^+^ T-cells from aging conventional or ICOSL^+/+^ NOD mice directly transfer diabetes into NOD.*scid* recipients without the need of prior *in vitro* activation. However, it has been observed that the transfer of diabetes is delayed by treatment of recipients mice by anti-class II monoclonal Abs (29), indicating that interactions between CD4^+^ effector T-cells and APCs through class II-mediated antigen presentation is still required for efficient transfer. In case of autoimmune neuromyopathy, the need for pre-activation of effector T-cells *in vitro* using anti-CD3 and anti-CD28-coated microplates prior to transfer has been reported when transferring autoimmune peripheral neuropathy by T lymphocytes from B7.2^−/−^ NOD donors (25). In order to address these issues in the present model, we investigated whether T-cells from ICOSL^+/+^ or ICOSL^−/−^ NOD donors could transfer diabetes or neuromyopathy to conventional NOD.*scid* or ICOSL^−/−^ NOD.*scid* recipients. As shown in Figure 4A, a significant delay was observed in transferring diabetes by CD4^+^ effector T-cells from 40-week-old control ICOSL^+/+^ into ICOSL^−/−^ as compared to ICOSL^+/+^ NOD.*scid* recipients. This was the case whatever CD4^+^T-cells were pre-activated *in vitro* by anti-CD3/anti-CD28 (Figure 4A) or not (Figure 4B). Strikingly, the transfer of diabetes by CD4^+^CD25^−^ T-cells was totally ineffective in ICOSL^−/−^ NOD.*scid* recipients, indicating that ICOS-ICOSL interaction is a prerequisite to the transfer of diabetes by naïve effector CD4^+^ T-cells from diabetic donors (Figure 4C). No significant delay was observed when transferring autoimmune neuromyopathy by pre-activated CD4^+^ effector T-cells from ICOSL^−/−^ NOD mice into ICOSL^−/−^ NOD.*scid* recipients as compared to ICOSL^+/+^ NOD.*scid* recipients (Figure 4D). We thus have evidence that ICOS^+^ T-cells from NOD ICOSL^−/−^ donors that have developed autoimmune neuromyopathy can transfer neuromyopathy into naïve ICOS^+/+^ICOSL^+/+^ NOD.*scid* as well as ICOS^+/+^ICOSL^−/−^ NOD.*scid* recipients.

## Discussion

The development of diabetes onto the NOD mouse background is associated with many gene variants that are scattered through the genome. Many of these susceptibility genes affect pathways involved in controlling immune responses (30–32). Among regions harboring these genes, one has been identified as carrying genes coding for T lymphocyte costimulatory molecules. A region on mouse chromosome 1 (*Idd5*) that carries costimulatory genes has been associated to diabetes in the NOD mouse (33). While the *Icos*^−/−^ strain contains a large non-NOD-derived interval on mouse chromosome 1 containing several genes encoding costimulatory molecules genes such as *Ctla4, Icos*, and *Cd28*, the *Icosl*^−/−^ strain genome contains almost only NOD alleles on all chromosomes and few NOD alleles around the candidate gene locus. In addition, this region does neither overlap with known *Idd* candidate regions or QTLs presently known to be involved in neuropathy (26). We therefore concentrated our phenotypical analysis on the *Icosl*^−/−^ strain rather than *Icos*^−/−^ strain.

Beyond inheritance of genes that predispose to general failure of immune-tolerance in the NOD mouse, some susceptibility regions habour genes that control the targeting or deviation of autoimmunity toward or from β-cells, respectively (34). The MHC class II I-A^g7^ allele that is associated with diabetes is a major gene that focuses autoimmunity on the islet (20, 24, 35). Beyond class II genes, other genes and environmental factors have been shown to deviate autoimmunity from β-cells toward other tissues (21, 23, 36). However, there has been so far no example of autoimmune deviation from β-cells toward skeletal muscles.

The diabetes protective phenotype observed in ICOS^−/−^ and ICOSL^−/−^ NOD mice thus indicates that the ICOS/ICOSL pathway is directly involved in the T cell activation observed in autoimmune diabetes. ICOSL is constitutively expressed on B cells, dendritic cells, and macrophages. It is not expressed on T-cells that, however, show lasting intrinsic alterations in transfer experiments. ICOS^+^T-cells that develop in an ICOSL^−^ environment maintain the capacity to induce a neuromyopathy even in an ICOSL sufficient environment. As previously reported, it is likely that a turning point in autoimmune activation in the NOD mouse takes place within a narrow window around 3 weeks of age (1, 37). That may apply to the activation of nerve- and muscle-specific T lymphocytes in ICOSL^−/−^ NOD mice. A striking observation in ICOSL^−/−^, as well as in ICOS^−/−^ NOD mice, is the deviation of autoimmunity from the islets toward muscles and peripheral nerves. T-cells are predominant among the different cell types observed within the infiltrates. Infiltrates of similar extent are observed in ICOS^−/−^ICOSL^−/−^ double-knockout NOD mice, which fit with previous reports of a single ICOS ligand. Finally, neuromuscular autoimmunity develops in ICOSL^−/−^ NOD mice within a time frame that is comparable to that of diabetes in conventional ICOSL^+/+^ NOD mice. However, the prevalence of neuromyopathy reaches 100% in female mice, which we never observed in case of diabetes in conventional female NOD mice.

Another example of the shift from diabetes to peripheral nerve autoimmunity has been evidenced in B7.2^−/−^ NOD mice in which myelin protein 0 has been identified as an auto-antigen (38). Autoimmune deviation in ICOSL^−/−^ NOD mice targets muscles and peripheral nerves, while it is restricted to peripheral neurons in B7.2^−/−^ NOD mice (14, 25). ICOS is expressed on T-cells after CD28/B7 engagement. We can suppose that, in absence of CD28/B7 signal, ICOS is weakly expressed in B7.2^−/−^ NOD mice and that the phenotype is partial, as compared to ICOSL^−/−^ NOD mice or ICOS^−/−^ NOD mice. A critical point is that the CD28/B7 pathway indeed remained unaltered in ICOS^−/−^ NOD mice. Autoimmune dilated cardiomyopathy has been reported following disruption of the negative immunoregulatory receptor *PD-1* in BALB/c mice along with diffuse deposition of IgG on the cardiomyocyte surface. Anti-troponin I autoantibodies have been identified as key effectors in this model (39, 40). Altogether, these observations point to local interactions of T-cells with resident cells as central in regulating organ-specific immune tolerance.

Finally, many diabetes auto-antigens that have been characterized so far are not strictly β-cell-specific and are expressed more generally in neuro-endocrine tissues. It has been reported that the glial sheath composed of peri-islet Schwann cells is destroyed in the pre-diabetic phase in the NOD mouse in which autoantibodies are detected and T-cells are activated against glial fibrillary acidic protein, which is predominantly expressed in Schwann cells and astrocytes (41). However, NOD mice do not spontaneously develop autoimmunity to nervous neuronal tissues.

Our data show that different mechanisms are involved in diabetes in NOD mice and in neuromuscular autoimmunity in ICOSL^−/−^ NOD mice. In the autoimmune neuromyopathy observed in ICOSL^−/−^ NOD mice, CD4^+^ T-cells are exclusive effectors as neuromuscular autoimmunity still develops in the absence of class I MHC molecules and thus of CD8^+^ effector T-cells. These data indicate that class I MHC molecules are not necessary for neuromyopathy development. This is in contrast with immune mechanisms involved in diabetes development in the conventional NOD mouse, in which the highest efficiency of diabetes transfer is observed when both CD4^+^ and CD8^+^ T-cells are co-injected into naive NOD recipients (3). MHC class II^−/−^ NOD mice and MHC class I^−/−^ NOD mice are both protected from diabetes (42, 43). It has further been shown that diabetes is induced in β2m^−/−^ NOD mice when class I expression is restored on β-cells (44). We previously showed in ICOS^−/−^ NOD mice that diabetes protection related with defective activation of effector T-cells. However, an accelerated form of diabetes was observed in BDC2.5 ICOS^−/−^ transgenic NOD mice, indicating both that diabetogenic BDC2.5 effectors were ICOS-independent and that the ICOS/ICOSL interaction was central in the activation of regulatory T-cells. Indeed, CD4^+^CD25^+^ regulatory T-cells from BDC2.5 ICOS^−/−^ NOD mice were significantly less efficient in suppressing the T cell response to a mimotope peptide than control regulatory T-cells *in vitro* (14). Similarly, we observed herein a decrease in relative and absolute numbers of CD4^+^FOXP3^+^ T-cells in SPL and pancreatic lymph nodes in ICOSL^−/−^ NOD mice. Cytokine patterns show that CD4^+^ T-cells present in muscle and nerve infiltrate of ICOSL^−/−^ NOD mice are biased toward a Th1 profile with high IFN-γ secretion. In this line, a previous study of inflammatory myopathies in patients evidenced the crucial role of ICOSL in inducing CD4^+^ T-cell responses (both Th1 and Th2), which could be suppressed *in vitro* by using an anti-ICOSL antibody (45).

Our transfer experiments show that neuromyopathy is efficiently transferred in NOD.*scid* recipients that express ICOSL or not. This suggests that neuromyopathogenic T-cells that have developed in the absence of ICOSL remain ICOSL-independent in the periphery, in contrast to diabetogenic T-cells that are ICOS-dependent. Therefore, naïve diabetogenic T-cells need ICOS/ICOSL signal to be activated and to transfer diabetes.

Events that link muscle and nerve autoimmunity remain an open issue in ICOS^−/−^ and ICOSL^−/−^ NOD mice. We never observed mice in which autoimmune responses to muscle and nerve were dissociated. One possibility that will require further assessment is that common auto-antigens are targeted by the autoimmune response in muscle and nerve. A related possibility is that distinct isoforms of the same auto-antigen are targeted. Damage in either muscles or nerves would then develop as a spreading of autoimmunity to epitope that would be shared with the primary auto-antigen against which the autoimmune response develops. This second possibility would require determining what tissue is the primary target of the autoimmune response that develops in the absence of ICOS/ICOSL signaling.

In conclusion, while ICOS^−/−^ or ICOSL^−/−^ NOD mice are protected from T1D, they develop autoimmunity against neural and muscular tissues, indicating that ICOS-ICOSL interactions are important in polarizing NOD autoimmunity. Neuromyopathy is CD4^+^/MHC class II-dependent and show a Th1 pattern. This model should be helpful for identifying new biomarkers in idiopathic forms of peripheral nerve and muscular autoimmunity and provides the first mouse model of spontaneous autoimmune myopathy akin to human myositis.

## Author Contributions

CBriet, NP, CM, OB, and CBoitard—substantial contributions to the conception and design of the work; CBriet, GB, IT, CBecourt, LD, J-CR, CM, OB, CBoitard, UR, and CA—acquisition, analysis, or interpretation of data for the work; CBriet, GB, IT, CBecourt, LD, J-CR, NP, CM, OB, CBoitard, UR, and CA—drafting the work or revising it critically for important intellectual content; CBriet, GB, IT, CBecourt, LD, J-CR, NP, CM, OB, CBoitard, UR, and CA—final approval of the version to be published and agreement to be accountable for all aspects of the work in ensuring that questions related to the accuracy or integrity of any part of the work are appropriately investigated and resolved.

## Conflict of Interest Statement

The authors declare that the research was conducted in the absence of any commercial or financial relationships that could be construed as a potential conflict of interest.

## Abbreviations

ALN, axillary lymph nodes; β2-m, beta-2-microglobulin; T1D, type 1 diabetes; ICOSL, ICOS ligand; MP0, myelin protein zero; PLN, pancreatic lymph nodes; PNS, peripheral nervous system, SPL, spleen; QTLs, quantitative trait loci.
